# Editorial: Establishing Genetic Pleiotropy to Identify Common Pharmacological Agents for Common Diseases

**DOI:** 10.3389/fphar.2019.01038

**Published:** 2019-09-13

**Authors:** Tracy A. O’Mara, Jyotsna Batra, Dylan Glubb

**Affiliations:** ^1^Molecular Cancer Epidemiology Laboratory, Genetics and Computational Biology Department, QIMR Berghofer Medical Research Institute, Brisbane, QLD, Australia; ^2^Cancer Program, School of Biomedical Sciences, Institute of Health and Biomedical Innovation, Queensland University of Technology, Brisbane, QLD, Australia

**Keywords:** pleiotropy, GWAS, endometrial cancer, *CYP19A1*, drug target, drug repurposing, pharmacogenetics

Pleiotropy, the phenomenon where one gene affects multiple traits, appears to be pervasive in biology. For example, findings from genome-wide association studies (GWAS) demonstrate that GWAS loci for different traits overlap nearly 50% of the time (Chesmore et al., 2018). Often these traits are related, as is illustrated by GWAS of endometrial cancer risk, where all 16 known loci contain variation that has been associated with other traits, including susceptibility to other cancer types as well as known risk factors for endometrial cancer (e.g., body mass index and age of menarche) (O’Mara et al., 2019).

Recently, it has been shown that drugs with genetically supported targets, including those identified by GWAS, are more likely to receive clinical approval than drugs whose targets are not supported by genetic evidence (Nelson et al., 2015; King et al., 2019). This effect is strongest when the causal gene underlying the genetic association has been established (e.g., Mendelian genes), highlighting an inherent problem with clinical translation of findings from GWAS, i.e., identifying the target genes of, primarily, non-coding genetic variation (Farashi et al., 2019; O’Mara et al., 2019). Even though a variety of bioinformatic and functional genomic approaches can be used for this purpose, and many data are publicly available (Pritchard et al.), this step appears to constitute a major bottleneck (Gallagher and Chen-Plotkin, 2018).

It has been estimated that 4,479 human genes encode proteins that are druggable, 1,427 of which are targets of drugs that either have been approved or are in clinical testing (Finan et al., 2017). In light of these and the observations above, the human genome may provide a source of effective targets for therapy. Furthermore, many public databases, using various types of evidence, can be queried to link genes to drugs or experimental compounds (Pritchard et al.). It is thus apparent that if a genetic study of disease identifies a target for which a drug already exists, then that drug may be able to be repurposed (or repositioned) to treat or serve as co-therapy to the new indication. Drug repurposing is not without caveats, though. The repurposed drug may still need to undergo pre-clinical validation (Pritchard et al.) before the repurposing is tested clinically, especially if there is limited evidence to demonstrate that the drug affects the function of the target. Also, issues with the patentability mean drug repurposing may be of less interest to pharmaceutical companies (Pritchard et al.).

As well as providing further indications for drug repurposing, pleiotropic drug targets may offer additional support for targeting. An example of such a pleiotropic target is provided by *CYP19A1*. Endometrial cancer GWAS risk variation at the *CYP19A1* gene also associates with circulating estradiol, which itself is causal for endometrial cancer (Thompson et al., 2016). *CYP19A1* encodes aromatase, the rate-limiting enzyme in the production of estradiol, which is targeted by aromatase inhibitors (used in the treatment of breast cancer). These drugs have been repurposed to treat endometrial cancer with some evidence of benefit for early-stage but not advanced disease (reviewed in Gao et al., 2014). However, it is possible that genetic markers could be used to identify patients with advanced disease who may receive benefit. Pharmacogenetic studies of aromatase inhibitors in breast cancer suggest that genetic variation in linkage disequilibrium with the pleiotropic *CYP19A1* GWAS variation may associate with better outcomes in advanced disease (Glubb et al.), though further studies are required to validate this finding.

Pharmacokinetic-related genes (i.e. those involved with drug absorption, distribution, metabolism, or excretion) are very often pleiotropic because they can affect traits related to exposure to multiple drugs or xenobiotics. Two examples are *CYP2C19* and *SLCO1B1*, which are associated with drug metabolism and transport, respectively. Both genes are highly pleiotropic with *CYP2C19* and *SLCO1B1* having been associated with responses to large numbers of drugs (588 for *CYP2C19* and 56 for *SLCO1B1*) (Cacabelos et al., 2019), including the pharmacokinetic profile of the antiplatelet agent ticagrelor as described in the current Research Topic (Zhu et al.) Further analysis of these two genes, and their pharmacogenetic variants, in phenome-wide association studies demonstrates that their pleiotropy may extend to the risk of common disease (Oetjens et al., 2016).

*TRIB3* is another pleiotropic gene with potential to inform drug therapy. A *TRIB3* missense variant (rs2295490) has been associated with early-onset type 2 diabetes, higher glucose levels in healthy controls (Prudente et al., 2009), and reduced risk of vascular events among type 2 diabetes patients with good glucose control (He et al., 2016). A study of patients with essential hypertension, presented in this Research Topic, indicates that rs2295490 may also associate with better vascular outcomes for patients treated with calcium channel blockers, angiotensin blockers, and α, β-adrenoceptor antagonists (Zhou et al.).

In conclusion, it is evident that pleiotropy offers the potential to identify drugs to treat common disease, and pleiotropic targets have advantages, particularly over those with no genetic basis. Moreover, establishing pleiotropy may provide an approach to prioritize targets for evaluation and identify genes that also affect clinical drug responses.

## Author Contributions

DG drafted the manuscript. All authors contributed to manuscript revision and approved the submitted version for publication.

## Funding

TO’M and JB are supported by National Health and Medical Research Council (NHMRC) Early Career (APP1090505) and Career Development Fellowships, respectively.

## Conflict of Interest Statement

The authors declare that the research was conducted in the absence of any commercial or financial relationships that could be construed as a potential conflict of interest.

## References

[B1] Cacabelos R. Cacabelos N. Carril J. C. (2019). The role of pharmacogenomics in adverse drug reactions. Expert Rev. Clin. Pharmacol. 12 (5), 407–442. 10.1080/17512433.2019.1597706 30916581

[B2] Chesmore K. Bartlett J. Williams S. M. (2018). The ubiquity of pleiotropy in human disease. Hum. Genet. 137 (1), 39–44. 10.1007/s00439-017-1854-z 29164333

[B3] Farashi S. Kryza T. Clements J. Batra J. (2019). Post-GWAS in prostate cancer: from genetic association to biological contribution. Nat. Rev. Cancer 19 (1), 46–59. 10.1038/s41568-018-0087-3 30538273

[B4] Finan C. Gaulton A. Kruger F. A. Lumbers R. T. Shah T. Engmann J. (2017). The druggable genome and support for target identification and validation in drug development. Sci. Transl. Med. 9 (383), 1–15. 10.1126/scitranslmed.aag1166 PMC6321762 28356508

[B5] Gallagher M. D. Chen-Plotkin A. S. (2018). The post-GWAS era: from association to function. Am. J. Hum. Genet. 102 (5), 717–730. 10.1016/j.ajhg.2018.04.002 29727686 PMC5986732

[B6] Gao C. Wang Y. Tian W. Zhu Y. Xue F. (2014). The therapeutic significance of aromatase inhibitors in endometrial carcinoma. Gynecol. Oncol. 134 (1), 190–195. 10.1016/j.ygyno.2014.04.060 24811574

[B7] He F. Liu M. Chen Z. Liu G. Wang Z. Liu R. (2016). Assessment of human tribbles homolog 3 genetic variation (rs2295490) effects on type 2 diabetes patients with glucose control and blood pressure lowering treatment. EBioMedicine 13, 181–189. 10.1016/j.ebiom.2016.10.025 27793583 PMC5264271

[B8] King E. A. Davis J. W. Degner J. F. (2019). Are drug targets with genetic support twice as likely to be approved? Revised estimates of the impact of genetic support for drug mechanisms on the probability of drug approval. bioRxiv 1–11, 513945 10.1101/513945 PMC6907751 31830040

[B9] Nelson M. R. Tipney H. Painter J. L. Shen J. Nicoletti P. Shen Y. (2015). The support of human genetic evidence for approved drug indications. Nat. Genet. 47 (8), 856–860. 10.1038/ng.3314 26121088

[B10] O’Mara T. A. Glubb D. M. Kho P. F. Thompson D. J. Spurdle A. (2019). Genome-wide association studies of endometrial cancer: latest developments and future directions. Cancer Epidemiol. Biomarkers Prev. 28 (7), 1095–1102. 10.1158/1055-9965.EPI-18-1031 31040137

[B11] Oetjens M. T. Bush W. S. Denny J. C. Birdwell K. Kodaman N. Verma A. (2016). Evidence for extensive pleiotropy among pharmacogenes. Pharmacogenomics 17 (8), 853–866. 10.2217/pgs-2015-0007 27249515 PMC5352965

[B12] Prudente S. Scarpelli D. Chandalia M. Zhang Y. Y. Morini E. Del Guerra S. (2009). The TRIB3 Q84R polymorphism and risk of early-onset type 2 diabetes. J. Clin. Endocrinol. Metab. 94 (1), 190–196. 10.1210/jc.2008-1365 18984671 PMC2630876

[B13] Thompson D. J. O’Mara T. A. Glubb D. M. Painter J. N. Cheng T. Folkerd E. (2016). CYP19A1 fine-mapping and Mendelian randomization: estradiol is causal for endometrial cancer. Endocr. Relat. Cancer 23 (2), 77–91. 10.1530/ERC-15-0386 26574572 PMC4697192

